# Dual PET Imaging with [^68^Ga]Ga-DOTA-TOC and [^18^F]FDG to Localize Neuroendocrine Tumors of Unknown Origin

**DOI:** 10.3390/curroncol32090497

**Published:** 2025-09-05

**Authors:** Ali Zaidi, Pavithraa Ravi, Ingrid Bloise, Sara Harsini, Heather C. Stuart, Hagen F. Kennecke, Ian Alberts, François Bénard, Don Wilson, Patrick Martineau, Jonathan M. Loree

**Affiliations:** 1 BC Cancer, Vancouver, BC V5Z 4E6, Canada; syed.zaidi@bccancer.bc.ca (A.Z.); pavithraa.ravi@bccancer.bc.ca (P.R.); sara.harsini@bccancer.bc.ca (S.H.); ian.alberts@bccancer.bc.ca (I.A.); fbenard@bccrc.ca (F.B.); dwilson@bccancer.bc.ca (D.W.); patrick.martineau@bccancer.bc.ca (P.M.); 2 Vancouver General Hospital, Vancouver, BC V5Z 1N1, Canada; heather.stuart@vch.ca; 3 Department of Medicine, Oregon Health & Science University, Portland, OR 97239, USA; kennecke@ohsu.edu

**Keywords:** neuroendocrine tumors, unknown primary, [^18^F]FDG PET/CT, [^68^Ga]Ga-DOTA PET/CT, carcinoid, gallium imaging, FDG

## Abstract

Neuroendocrine tumors of unknown primary (CUP-NET) present a diagnostic challenge when conventional imaging fails to localize the primary tumor. This study aimed to evaluate the diagnostic value of concurrent [^68^Ga]Ga-DOTA-TOC and [^18^F]FDG PET/CT imaging in localizing primary tumors in patients with histologically confirmed CUP-NET. Thirty-four patients underwent both imaging modalities as part of a prospective imaging protocol after negative conventional imaging or [^111^In]In-octreotide scintigraphy. Primary tumor detection rates were assessed, and imaging characteristics compared between the two modalities. The overall localization rate was 58.9% (20/34). Of these, 90% (18/20) of primary tumors were identified solely by [^68^Ga]Ga-DOTA-TOC PET/CT, with the remaining two visualized by both modalities. [^18^F]FDG PET/CT did not independently localize any primary tumors. Identified primaries were limited to grade 1 (60%) or grade 2 (40%) tumors, predominantly in the small intestine (95%). Among localized cases, 45% (9/20) underwent surgical resection and 15% (3/20) became eligible for peptide receptor radionuclide therapy. [^68^Ga]Ga-DOTA-TOC PET/CT demonstrated superior detection of metastatic lesions compared to [^18^F]FDG PET/CT (97.1% vs. 70.6%, *p* = 0.006). No significant survival differences were observed between patients with localized versus non-localized primaries. These findings support the value of [^68^Ga]Ga-DOTA-TOC PET/CT for identifying primary tumors in CUP-NET. Further research is warranted to explore the role of [^18^F]FDG PET/CT in high-grade NETs.

## Simple Summary

Patients with neuroendocrine cancers sometimes have tumors that spread without doctors being able to find where the cancer started. This is called cancer of unknown primary (CUP). Finding the primary tumor is important because it helps guide treatment decisions. In this study, we used two specialized imaging scans—[^68^Ga]Ga-DOTA-TOC PET/CT and [^18^F]FDG PET/CT—to look for the source of cancer in patients whose standard scans had been negative. We found that [^68^Ga]Ga-DOTA-TOC PET/CT could successfully locate the hidden primary tumor in around 60% of patients, while [^18^F]FDG PET/CT alone did not help find any independently. Most tumors that were found started in the small intestine and were lower grade cancers. Finding the primary allowed some patients to have surgery or targeted treatments. These results show that [^68^Ga]Ga-DOTA-TOC PET/CT is a valuable tool to help locate hidden tumors in this rare patient group.

## 1. Introduction

Cancer of unknown primary (CUP) refers to histologically confirmed metastatic cancers with an unidentified primary site [1]. Despite accounting for less than 5% of CUPs, approximately 13% of neuroendocrine tumors (NETs) have an undetermined primary site at the time of diagnosis (CUP-NET) [2]. CUP-NETs have a 10-year survival rate of less than 20% [3]. Knowledge of the primary site allows oncologists to prioritize treatment sequencing and may have implications for surgical management. Surgical resection of locoregional disease is the only curative treatment for NETs but debulking surgery may be considered in metastatic NETs to improve survival [4,5,6,7,8]. Accurate diagnosis and tumor localization are therefore critical for prognostication, therapeutic decision making, and improved survival.

Recent advances in the diagnosis of NETs have introduced several promising tools [9]. Expanded immunohistochemical panels and refined pathological classifications have improved diagnostic accuracy and consistency across institutions [10]. Newer positron emission tomography (PET) tracers such as [^64^Cu]Cu-labeled somatostatin analogs and somatostatin receptor antagonists may offer higher sensitivity and more flexible logistics than conventional methods [11,12]. Emerging non-invasive technologies, including circulating biomarkers and AI-based radiomics, are also being explored and may aid in the evaluation of CUP-NETs in the future [13,14]. While these developments highlight the evolving diagnostic landscape of NETs, their current utility remains limited, and for CUP-NETs the challenge of localizing the primary site continues to rely largely on imaging-based strategies.

Traditional imaging modalities such as computed tomography (CT) and magnetic resonance imaging (MRI) often do not localize the primary site in CUP-NETs [15]. NETs are characterized by the overexpression of somatostatin receptors (SSTRs), making them a unique target for receptor-based imaging using radiolabeled somatostatin analogs. However, somatostatin receptor imaging with [^111^In]In-octreotide scintigraphy has limited utility in the primary site detection of CUP-NETs with rates of only 8–39% [16,17]. PET-based somatostatin imaging has a higher sensitivity and specificity than [^111^In]In-octreotide in detecting NET lesions [17,18,19]. Combined [^18^F]FDG and SSTR-PET/CT can be performed [20] but the role and ideal population for dual imaging are unclear.

A subset of well-differentiated NETs can also exhibit intense FDG uptake [21]. However, its added value in the setting of CUP-NET is unclear with no evidence that dual-tracer PET/CT results in enhanced primary identification. Previous studies have explored the utility of [^68^Ga]Ga-DOTA-based imaging in CUP-NETs [17,22,23,24,25,26,27,28,29,30,31,32], but data on the utility of concurrent [^68^Ga]Ga-DOTA-TOC and [^18^F]FDG PET/CT remains limited in comparison [33,34]. In this study, we aimed to evaluate the diagnostic value of FDG and DOTA PET when used concurrently in the localization of CUP-NETs.

## 2. Materials and Methods

### 2.1. Study Population

In the province of British Columbia (BC), Canada, all [^68^Ga]Ga-DOTA-based imaging is performed on a prospective protocol (NCT03583528) that also includes the performance of an [^18^F]FDG PET/CT. Thirty-four patients who underwent dual [^68^Ga]Ga-DOTA-TOC and [^18^F]FDG PET/CT from December 2018-February 2023 after histopathological confirmation of a metastatic NET of unknown origin were included in this study. Inclusion criteria were: (i) histopathological confirmation of metastatic NET; (ii) absence of a localized primary site on conventional imaging (CT and/or MRI) and [^111^In]In-Octreotide scintigraphy; and (iii) having undergone both [^68^Ga]Ga-DOTA-TOC and [^18^F]FDG PET/CT within the study period. Patients were excluded if the pathology was not consistent with a CUP-NET or the primary site had been localized by conventional imaging prior to dual PET/CT. The study was approved by the BC Cancer Institutional Review Board and performed in accordance with the Declaration of Helsinki and our institution’s ethics guidelines and protocol. Informed consent was obtained for all participants prior to their inclusion in the study. Consent for publication was obtained from all participants.

### 2.2. [^68^Ga]Ga-DOTA-TOC and [^18^F]FDG Preparation

Fully automated labelling of [^68^Ga]Ga-DOTA-TOC was performed using a labelling system with ^68^Ge/^68^Ga generators (Isotopes Technologies Garching Gmbh (ITG), Garching, Germany; iThemba LABS, Cape Town, South Africa), and peptide reagent kits, sterile cassettes and GMP grade DOTA-TOC acetate vials from BC Cancer (Vancouver, BC, Canada). Each patient received an intravenous bolus injection of [^68^Ga]Ga-DOTA-TOC in a dose determined by body weight and varying between 90–220 MBq with an average dose of 185 MBq.

[^18^F]FDG was prepared and administered as per institutional standard of care with subjects receiving an intravenous bolus injection of a dose determined by body weight and varying between 237–521 MBq with an average dose of 296 MBq.

### 2.3. PET/CT Imaging

PET/CT images were obtained approximately 60 min post injection using hybrid 3D PET/CT scanners (Discovery 690 or Discovery MI, GE Healthcare, Waukesha, WI, USA). The field of view extended from the eyes to the mid-thighs. Images were reconstructed using iterative techniques with a minimal matrix size of 192 × 192 for PET. PET, CT, and fused PET/CT images were analyzed in three orthogonal planes using attenuated and non-attenuation corrected PET data using HybridViewer PET-CT fusion software (version 2.16.0.2, Hermes Medical Solutions). The CT component consisted of a low-dose, unenhanced scan for attenuation correction and anatomic localization, with 3 mm slices and additional 1.25 mm thin cuts through the lungs. For clinical correlation, most patients (28/34) also underwent a diagnostic contrast-enhanced CT scan within six months of the PET/CT.

### 2.4. Visual Analysis

All studies were reviewed and reported by experienced nuclear medicine physicians at a single provincial institution providing all PET/CT imaging during the study period. A total of 14 different reporting physicians contributed across PET/CT studies. All reported standardized uptake values (SUVs) consisted of maximum SUVs measured within a region of interest. Individual lesions were defined as foci of radiotracer uptake significantly greater than background and visually distinct from the normal biodistribution of the radiotracer. Krenning scores, lesion location and total number of lesions were obtained for each study. Mesenteric lymph node involvement was specifically recorded, and a sensitivity analysis was performed classifying patients with mesenteric nodal metastases as having an occult small bowel primary when a discrete lesion was not directly visualized.

### 2.5. Statistical Analysis

Demographic, imaging and clinical characteristics of the study cohort were summarized. Continuous variables were reported as mean ± standard deviation (SD) or median (range) depending on distribution. Categorical variables were reported using counts and percentages. Patients were categorized based on primary site localization status for the purpose of analysis. Intergroup comparisons were analyzed using the Mann–Whitney U Test for non-parametric continuous variables and chi-square tests, including Fisher’s exact test when applicable, for categorical variables. Spearman rank correlation coefficient (r) was measured to determine the association between the maximum standardized uptake value (SUVmax) of the primary site on [^68^Ga]Ga-DOTA-TOC PET/CT and size of the localized primary tumor. The log-rank test was used to compare survival distributions between localization groups. Statistical analysis was performed on GraphPad Prism (version 10.0.1 for Mac OS X, GraphPad Software, San Diego, CA, USA). *p* < 0.05 was considered statistically significant.

## 3. Results

### 3.1. Baseline Characteristics

Baseline characteristics are found in Table 1. Median age at diagnosis was 68 years (interquartile range, 60–73) with 55.9% female patients. No significant difference was observed between localization status for sex (*p* = 0.127) and age (*p* = 0.752). The most common metastatic sites of biopsy were the liver (76.5%), lymph nodes (20.6%) and breast (2.9%), with all reported tumor morphology being well-differentiated. Over half of all patients (18/34; 52.9%) were classified as grade 2 (G2) NETs. Previous [^111^In]In-octreotide imaging detected metastatic lesions on at least one scan in 91.2% of patients overall (31/34; 95% confidence interval [95%CI], 76.3–97.8%) with no significant difference between those with/without their primary localized (*p* = 0.556).

### 3.2. Primary Tumor Detection by PET/CT

Time between diagnosis of CUP-NET and the first PET/CT scan ranged from 17 days to over 8 years (3206 days) and the median number of days was significantly longer in the group where the primary was not identified (*p* = 0.003). The overall primary site detection rate using concurrent [^68^Ga]Ga-DOTA-TOC and [^18^F]FDG PET/CT was 58.8% (20/34; 95%CI, 42.2–73.7%). A comparison of patients with a primary site identified on PET/CT is summarized in Table 2. Of these 20 patients with a localized primary, 9 (45%) underwent surgical resection providing histological confirmation and the remaining 11 (55%) were determined based on clinician assessment and imaging follow-up. [^68^Ga]Ga-DOTA-TOC PET/CT was the only localizing modality in 90% (18/20) of patients, while the primary lesion was visualized on both [^18^F]FDG and [^68^Ga]Ga-DOTA-TOC PET/CT in the remaining two subjects. No patients had a primary identified only because of [^18^F]FDG PET/CT. In a sensitivity analysis accounting for mesenteric lymph node involvement as an indicator of an occult small bowel primary, one additional patient was reclassified as having a localized primary (21/34; 61.8%).

Among patients with a localized primary tumor on PET/CT, grade 1 (G1) NETs accounted for 60.0%. G2 NETs were predominant in patients without a localized primary (71.4% of group). Grade 3 (G3) NETs were exclusively observed in patients without a localized primary, representing 14.3% of this group and 5.9% of the total cohort.

All primary sites were found in either the small intestine (95%), or stomach (5%) (Table 3). Primary tumors had a mean [^68^Ga]Ga-DOTA-TOC PET/CT SUVmax of 15.7 (SD, ±9.0) and a mean lesion size of 14.8 mm (SD, ±3.4). There was no correlation between [^68^Ga]Ga-DOTA-TOC PET/CT SUVmax of the primary site and tumor size on imaging (r = 0.22; *p* = 0.35).

### 3.3. Comparison of PET/CT Modalities for Metastatic Disease

A comparison of imaging characteristics between the two modalities and their ability to detect metastases is shown in Table 4. [^68^Ga]Ga-DOTA-TOC PET/CT exhibited a significantly higher positive scan rate, including both locoregional and metastatic disease, compared to FDG for the overall cohort (97.1% vs. 70.6%; *p* = 0.006) and within the subgroup of patients with localized primary sites (100% vs. 60.0%; *p* = 0.003). In the subgroup of patients in which dual functional imaging did not localize a primary lesion (n = 14), [^68^Ga]Ga-DOTA-TOC PET/CT failed to detect any metastatic sites in just one subject. A significant association was found between the number of detected lesions and the radiotracer, both overall (*p* = 0.001) and in the identified primary site subgroup (*p* = 0.001). [^68^Ga]Ga-DOTA-TOC PET/CT found more liver metastases (26/34, 76.4% vs. 14/34, 41.2%; *p* = 0.003) and regional nodes (17/34, 50.0% vs. 5/34, 14.7%; *p* = 0.001) with no significant difference for detection of bone metastases (9/34, 26.5% vs. 4/34, 11.8%; *p* = 0.123), and lung lesions (2/34, 5.9%; *p* = 1.0).

A trend towards a higher median [^68^Ga]Ga-DOTA-TOC PET/CT Krenning score was observed in patients with an identified primary site on PET/CT (4 vs. 3, *p* = 0.075). Two out of 14 patients without an identified primary were initially suggested to have a primary site found on PET/CT imaging but could not be confirmed on follow-up clinical investigations.

### 3.4. Treatment and Survival Implications Following Imaging

Based on the findings, 45% (9/20) underwent surgical resection to remove the primary site and one patient (1/20; 5.0%) was still under assessment for resection at the time of data collection and had not undergone surgery. After surgery, 44.4% of primary tumors were classified as G2 NETs (4/9) and 22.2% (2/9) were given a higher grade compared to initial diagnosis, with the highest increase from G1 to G2. Among the ten remaining patients who did not undergo surgical resection, 3 (30.0%) had a primary tumor identified in a site eligible for peptide receptor radionuclide therapy (PRRT) reimbursement in our province, which they otherwise would not have qualified for. Of these three patients, two received at least one dose of PRRT. Furthermore, 2/10 (20.0%) started a new somatostatin analog (SSA) regimen, while the remaining 6/10 (60.0%) continued the same therapy.

**Figure 1:**
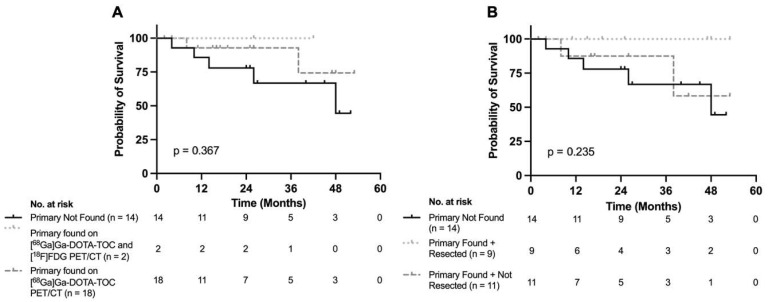
Kaplan–Meier Survival Analysis by Localization and Resection Status in CUP-NET Patients. Kaplan–Meier survival of CUP-NET patients by (**A**) localization status and modality and (**B**) primary tumor resection status. The log-rank test was used to determine statistical significance. Sample sizes for each group are indicated in the figure.

In a comparison of survival outcomes (Figure 1), patients with a detected primary lesion (*p* = 0.367) and a resected primary tumor (*p* = 0.235) showed no difference in overall survival compared to patients without a detected primary.

## 4. Discussion

In this study, we explored the value of concurrent [^68^Ga]Ga-DOTA-TOC and [^18^F]FDG PET/CT imaging in a prospective cohort of histologically confirmed CUP-NET patients who were not localized on previous [^111^In]In-octreotide and CT/MRI imaging. NETs are a heterogeneous group of neoplasms exhibiting a wide spectrum of clinical behaviors and presentations [10]. As surgery is considered the only opportunity for cure for patients with locoregional disease, defining the primary tumor is important in disease management. In the setting of metastatic disease, identifying the primary tumor can guide surgical management if debulking is being considered and facilitate appropriate medical management for unresectable disease [2,35]. The role of [^68^Ga]Ga-DOTA PET/CT in primary tumor localization has been described [36], but given the variability in NET tumor biology it is important to consider how functional imaging can reflect biology using alternate radiotracers. Our study found comparable detection rates with previous literature for [^68^Ga]Ga-DOTA-TOC PET/CT, but a lower detection rate was observed in [^18^F]FDG PET/CT, and all FDG-identified tumors were also detected by [^68^Ga]Ga-DOTA-TOC PET/CT. This suggests low utility of [^18^F]FDG PET for the localization of unknown primaries.

Concurrent [^68^Ga]Ga-DOTA-TOC PET/CT and [^18^F]FDG PET/CT is of unclear utility in the management of NETs, and regional practice patterns and reimbursement often dictate selection of patients for dual functional imaging. Our clinical trial provides a unique opportunity to review the utility of [^18^F]FDG PET in CUP-NETs in an unbiased population as all patients undergoing functional imaging in our province receive both scans. Importantly, dual-tracer PET has been evaluated in only a limited number of studies in patients with CUP-NET. Sampithirao & Basu previously examined dual-tracer [^68^Ga]Ga-DOTA-TATE and [^18^F]FDG PET/CT, but their population did not include patients who had previously undergone [^111^In]In-octreotide imaging [33]. In another study by Chen et al. using dual functional imaging with [^68^Ga]Ga-DOTA-TOC and [^18^F]FDG PET/CT, only 10 patients in their cohort had histologically proven metastatic NETs with negative localization on conventional imaging, with the remainder being clinically suspected NETs [34]. Our study, with a larger uniformly defined cohort of histologically confirmed CUP-NETs and inclusion of patients with prior [^111^In]In-octreotide and CT/MRI imaging, adds to our understanding of the utility of dual functional imaging in CUP-NETs.

Our study offers novel contributions compared with prior reports. Its prospective design and inclusion of all patients undergoing dual functional imaging provincially provide a more pragmatic estimate of detection rates in real-world practice. The focus on patients with negative localization on previous [^111^In]In-octreotide imaging allows for a clearer assessment of the added value of dual PET in situations where standard approaches fail to localize the primary site. Histopathological confirmation of metastatic NET across our cohort strengthens the internal validity of the findings. Taken together, these features allow our study to more precisely characterize the added value of dual-tracer PET in a uniquely defined cohort of CUP-NET patients while complementing earlier studies.

An overall detection rate of 58.8% (20/34, 95%CI 42.2–73.7%) was yielded using [^68^Ga]Ga-DOTA-TOC PET/CT and [^18^F]FDG PET/CT. Specifically, 18/20 CUP-NETs were detected by [^68^Ga]Ga-DOTA-TOC PET/CT only, while the remaining two were detected by both modalities. Several previous studies have explored the efficacy of [^68^Ga]Ga-DOTA imaging alone. The three most common analogs are [^68^Ga]Ga-DOTA-TOC, [^68^Ga]Ga-DOTA-TATE and [^68^Ga]Ga-DOTA-NOC, with all three exhibiting a high affinity for SSTR subtype 2 [37]. Specific detection rates for [^68^Ga]Ga-DOTA-TOC PET/CT have ranged from 38% [32], to 50% [28] for CUP-NETs in previous studies. Higher detection rates were noted in the other analogs including 59–60% for [^68^Ga]Ga-DOTA-NOC [23,25,29] and 29–79% for [^68^Ga]Ga-DOTA-NOC [24,27,30,31]. A meta-analysis by De Dosso et al. including the abovementioned studies found a pooled detection rate of 56% (95% CI, 48–63%), which is consistent with our findings [38]. Additional subgroup analysis comparing the three analogs did not find a significant difference in detection rates.

We note that mesenteric lymph node metastases may suggest a small bowel primary even when a discrete lesion is not visualized [39]. In a sensitivity analysis, reclassifying one patient with mesenteric nodal metastases as having an occult small bowel primary did not significantly change the cohort characteristics. However, this observation highlights that PET/CT imaging can be aided by patterns in nodal involvement to help determine the likely primary site, which should be considered in interpretation.

Only two patients in our cohort had their primary site identified on [^18^F]FDG PET/CT, both of which were also identified on [^68^Ga]Ga-DOTA-TOC PET/CT. This detection rate of 5.9% is notably lower than the rates of 13.7% [33] and 23.5% [34] observed with dual-tracer PET in previous literature. However, this discrepancy could be attributed to all patients in our cohort having well-differentiated tumors with the majority (94.1%) G1 and G2 grade NETs. Poorly differentiated neuroendocrine carcinomas (NECs), which typically demonstrate higher [^18^F]FDG avidity, were not represented. Well-differentiated G1 and G2 NETs display low metabolic activity, making it difficult for [^18^F]FDG PET/CT to detect lesions, and suggest limited diagnostic value for the majority of CUP-NET patients. Kayani et al. previously observed significantly higher uptake of [^18^F]FDG over [^68^Ga]Ga-DOTA-TATE in G3 patients while the opposite was observed in G1 patients [20]. This suggests [^18^F]FDG PET/CT may be most informative in higher-grade, poorly differentiated disease rather than the well-differentiated, low and intermediate grade CUP-NET setting.

Ninety-five percent of the localized primary tumors in our cohort originated from the small intestine. This proportion was higher than previous studies, with Nakamoto et al. reporting 86% in a smaller cohort of only 14 patients [28]. Among studies with at least 30 patients, Pruthi et al. reported the highest proportion of 66% (27/41) small intestine NETs [29]. Our study aligns closer with previous surgical exploration studies where a small intestine primary site was identified in 69.8% [40] and 86.4% [41] of patients, respectively. In another study among CUP-NET patients with liver metastases, all localized primary tumors were in the small intestine [15]. We also examined the association between SUVmax in primary lesions and the size on [^68^Ga]Ga-DOTA-TOC PET/CT and found a weak Spearman rank correlation of 0.223. To our knowledge, this relationship has not previously been explored in the context of NETs and [^68^Ga]Ga-DOTA imaging, but could highlight a potential biological relationship that warrants validation in independent datasets.

While no statistically significant difference in overall survival was observed between patients with and without primary localization (Figure 1), this finding should be interpreted with caution. The relatively small cohort size and further stratification by localization and/or resection status limited statistical power, although a trend toward improved outcomes was noted. Moreover, as all patients presented with metastatic disease, overall survival is likely driven more by disease burden than by primary localization alone. Surgical resection is the only curative treatment for NETs, and debulking surgery can prolong survival in metastatic cases [4,5,6,7,8]. However, in the CUP-NET setting, patients often present with extensive disease that limits surgical intervention even when the primary site is identified. Larger studies may be better positioned to further ascertain the prognostic significance of primary tumor identification in CUP-NETs.

The study must be considered in the context of limitations. While patient data were collected prospectively, it is still a small population of patients that were evaluated. Although all functional imaging was reviewed by expert nuclear medicine physicians, scans were interpreted by multiple readers without inter-operator verification. This could introduce minor variability, but reflects real-world practice. Additionally, not all patients underwent histological confirmation of their localized primary sites if it was not feasible or radiographic evidence was deemed conclusive, which can potentially introduce a degree of uncertainty in the final results. Despite these limitations, our study still represents one of the largest cohorts of patients receiving both PET scans and they were obtained in a relatively unbiased fashion rather than performing [^18^F]FDG PET/CT only in select cases based on unvalidated criteria.

## 5. Conclusions

Our study highlights the utility of [^68^Ga]Ga-DOTA-TOC PET/CT in localizing primary lesions in well-differentiated, low and intermediate grade CUP-NETs previously negative on [^111^In]In-octreotide scintigraphy. We found limited added value of [^18^F]FDG PET/CT, as it did not contribute to identifying primary lesions beyond what was achieved with [^68^Ga]Ga-DOTA-TOC PET/CT. This suggests that [^18^F]FDG PET/CT may have limited utility in primary location determination. Further research is needed to clarify the role of [^18^F]FDG PET/CT in prognostication and imaging of high-grade and poorly differentiated CUP-NET patients.

## Figures and Tables

**Table 1:** Baseline Characteristics.

Primary Site Localization Status	All (*n* = 34)	Yes (*n* =20)	No (*n* = 14)	*p*
**Sex, n (%)**				0.127
* Female*	19 (55.9)	9 (45.0)	10 (71.4)	
**Median Age at Diagnosis (Range)**	68 (28–86)	66.5 (35–85)	69 (28–86)	0.752
**Median Days from Diagnosis to First PET Scan (Range)**	275 (17–3206)	181 (17–2894)	1575 (78–3206)	0.003
**Median Days between DOTA-TOC and Octreotide Scans (Range)**	150 (23–1654)	103.5 (23–610)	168.5 (43–1654)	0.087
**Median Days between DOTA-TOC and FDG scans (Range)**	4 (1–48)	4 (1–48)	5 (1–11)	0.704
**Octreotide Positive Scan Rate for Metastases, n (%)**	31 (91.2)	19 (95.0)	12 (85.7)	0.556
**Metastatic Site of Biopsy, n (%)**				
* Liver*	26 (76.5)	15 (75.0)	11 (78.6)	
* Lymph Node*	7 (20.6)	5 (25.0)	3 (21.4)	
* Breast*	1 (2.9)	0 (0.0)	1 (7.1)	
**Tumor Grade, n (%)**				
*Grade* 1	14 (41.2)	12 (60.0)	2 (14.3)	
*Grade* 2	18 (52.9)	8 (40.0)	10 (71.4)	
*Grade* 3	2 (5.9)	0 (0.0)	2 (14.3)	
**Tumor Differentiation, n (%)**				
* Well Differentiated*	34 (100)	20 (100)	14 (100)	
* Poorly Differentiated*	0 (0.0)	0 (0.0)	0 (0.0)	

“Yes” = primary tumor successfully localized by PET imaging; “No” = primary tumor not localized by PET imaging; DOTA-TOC = [^68^Ga]Ga-DOTA-TOC PET/CT; FDG = [^18^F]FDG PET/CT; *p*-values refer to comparisons between the “Yes” and “No” groups.

**Table 2:** Patient-Wide Summary of Management Changes after Primary Localization.

No.	Primary Site	Ki-67 (%)	DOTA-TOC Assessment	FDG Assessment	Localizing Modality	Metastasis	Surgical Resection
**1**	Small Intestine	5	+	−	DOTA-TOC	Liver	No
**2**	Small Intestine	NR	+	+	DOTA-TOC	Liver, LN,	Yes
**3**	Small Intestine	5	+	+	DOTA-TOC	Liver	Yes
**4**	Small Intestine	3	+	−	DOTA-TOC	Liver	Yes
**5**	Small Intestine	<2	+	+	Both	Liver, LN, Bone	No
**6**	Small Intestine	1–2	+	−	DOTA-TOC	LN	No
**7**	Small Intestine	1–2	+	+	DOTA-TOC	Liver, LN	No
**8**	Small Intestine	7	+	+	Both	Liver, LN, Bone	No
**9**	Small Intestine	2	+	−	DOTA-TOC	Liver, LN	Yes
**10**	Small Intestine	2	+	−	DOTA-TOC	Liver, LN	Yes
**11**	Small Intestine	<1	+	+	DOTA-TOC	Liver, LN	No
**12**	Small Intestine	<3	+	−	DOTA-TOC	Liver, LN, Bone	No
**13**	Small Intestine	<1	+	−	DOTA-TOC	Liver	Yes
**14**	Small Intestine	5	+	+	DOTA-TOC	Liver	Yes
**15**	Small Intestine	<1	+	+	DOTA-TOC	LN	Yes
**16**	Small Intestine	<1	+	+	DOTA-TOC	LN	Yes
**17**	Small Intestine	<3	+	+	DOTA-TOC	Liver	No
**18**	Stomach	12	+	+	DOTA-TOC	Liver, Spleen	No
**19**	Small Intestine	10	+	−	DOTA-TOC	Liver	No
**20**	Small Intestine	4	+	+	DOTA-TOC	LN, Bone, Lung	No

NR = not reported; LN = lymph node; DOTA-TOC = [^68^Ga]Ga-DOTA-TOC PET/CT; FDG = [^18^F]FDG PET/CT; + = positive uptake; − = no uptake. DOTA-TOC and FDG assessments include metastatic lesions.

**Table 3:** Imaging Characteristics of Primary Tumor Site.

Imaging Characteristic	Value
**Primary Site, n (%)**	
**Small Intestine**	19 (95.0)
* Ileum*	7 (36.8)
* Jejunum and ileum*	2 (10.5)
* Not specified*	9 (47.4)
**Stomach**	1 (5.0)
**DOTA-TOC SUV_max_, mean ± SD**	15.7 ± 9.0
**DOTA-TOC Lesion Size, mean ± SD (mm)**	14.8 ± 3.4

**Table 4:** Metastatic Lesion Imaging Comparison Between [^68^Ga]Ga-DOTA-TOC PET/CT and [^18^F]FDG PET/CT.

Primary Site Localization Status	All Patients with CUP-NET (n = 34)		*p*	CUP-NETs with a Primary Identified by PET/CT (n = 20)		*p*	No Primary Identified by PET/CT (n = 14)		*p*
	[^68^Ga]Ga-DOTA-TOC	[^18^F]FDG		[^68^Ga]Ga-DOTA-TOC	[^18^F]FDG		[^68^Ga]Ga-DOTA-TOC	[^18^F]FDG	
**Overall Scan Positivity, n (%) [95% CI]**	33 (97.1) [0.84–1.00]	24 (70.6) [0.54–0.83]	0.006	20 (100) [0.82–1.0]	12 (60.0) [0.36–0.81]	0.003	13 (92.9) [0.66–1.00]	12 (85.7) [0.59–0.97]	1.0
**Total Lesions, n (%)**			0.001			0.001			0.407
0	1 (2.9)	10 (29.4)		0 (0.0)	8 (40.0)		1 (7.1)	2 (14.3)	
1–5	11 (32.4)	15 (44.1)		7 (35.0)	8 (40.0)		4 (28.6)	7 (50.0)	
>5	22 (64.7)	9 (26.5)		13 (65.0)	4 (20.0)		9 (64.3)	5 (35.7)	
**Sites of Metastasis, n (%)**									
* Liver*	26 (76.4)	14 (41.2)	0.003	16 (80.0)	7 (35.0)	0.010	11 (78.6)	7 (50.0)	0.237
* LN*	17 (50.0)	5 (14.7)	0.001	11 (55.0)	2 (10.0)	0.006	6 (42.9)	3 (21.4)	0.420
* Bone*	9 (26.5)	4 (11.8)	0.123	3 (15.0)	1 (5.0)	0.605	6 (42.9)	3 (21.4)	0.420
* Lung*	2 (5.9)	2 (5.9)	1.0	0 (0.0)	0 (0.0)	1.0	2 (14.3)	2 (14.3)	1.0
**Median Krenning Score (Range)**	4 (0–4)	NA		4 (3–4)	NA		3 (0–4)	NA	

CUP-NET = cancer of unknown primary neuroendocrine tumor; DOTA-TOC = [^68^Ga]Ga-DOTA-TOC PET/CT; FDG = [^18^F]FDG PET/CT; CI = confidence interval; NA = not applicable; LN = lymph node; Krenning Score = somatostatin receptor expression score (range 0–4); lesion counts refer to the number of metastatic sites identified per patient.

## Data Availability

De-identified data from this study may be made available to collaborators via institutionally approved data transfer agreements, upon written request to the corresponding author. The present analysis is part of a larger clinical trial that has not yet had its primary publication, and therefore data cannot be publicly released at this time (clinicaltrials.gov ID: NCT03583528).

## Abbreviations

The following abbreviations are used in this manuscript:

NET	Neuroendocrine tumor
PET	Positron emission tomography
CT	Computed tomography
MRI	Magnetic resonance imaging
SUVmax	Maximum standardized uptake value
CUP-NET	Neuroendocrine tumor of unknown primary
CUP	Cancer of unknown primary
PRRT	Peptide receptor radionuclide therapy
SSTR	Somatostatin receptor
FDG	Fluoro-deoxy-glucose
LN	Lymph node
NEC	Neuroendocrine carcinoma

## References

[1] Pavlidis N., Pentheroudakis G. (2012). Cancer of Unknown Primary Site. Lancet.

[2] Pavel M., O’Toole D., Costa F., Capdevila J., Gross D., Kianmanesh R., Krenning E., Knigge U., Salazar R., Pape U.-F. (2016). ENETS Consensus Guidelines Update for the Management of Distant Metastatic Disease of Intestinal, Pancreatic, Bronchial Neuroendocrine Neoplasms (NEN) and NEN of Unknown Primary Site. Neuroendocrinology.

[3] Abdel-Rahman O. (2021). A Real-World, Population-Based Study for the Incidence and Outcomes of Neuroendocrine Neoplasms of Unknown Primary. Neuroendocrinology.

[4] Fendrich V., Bartsch D.K. (2011). Surgical Treatment of Gastrointestinal Neuroendocrine Tumors. Langenbeck’s Arch. Surg..

[5] Bertani E., Fazio N., Radice D., Zardini C., Spinoglio G., Chiappa A., Ribero D., Biffi R., Partelli S., Falconi M. (2017). Assessing the Role of Primary Tumour Resection in Patients with Synchronous Unresectable Liver Metastases from Pancreatic Neuroendocrine Tumour of the Body and Tail. A Propensity Score Survival Evaluation. Eur. J. Surg. Oncol. (EJSO).

[6] Bertani E., Fazio N., Radice D., Zardini C., Grana C., Bodei L., Funicelli L., Ferrari C., Spada F., Partelli S. (2016). Resection of the Primary Tumor Followed by Peptide Receptor Radionuclide Therapy as Upfront Strategy for the Treatment of G1–G2 Pancreatic Neuroendocrine Tumors with Unresectable Liver Metastases. Ann. Surg. Oncol..

[7] Polcz M., Schlegel C., Edwards G.C., Wang F., Tan M., Kiernan C., Solórzano C.C., Idrees K., Parikh A., Bailey C.E. (2020). Primary Tumor Resection Offers Survival Benefit in Patients with Metastatic Midgut Neuroendocrine Tumors. Ann. Surg. Oncol..

[8] Hallet J., Law C., Hallet J., Law C., Pasieka J., Koea J., Meyer-Rochow W., the Commonwealth Neuroendocrine Tumours Research Collaborative (CommNETs) Surgical Section (2021). Role of Primary Tumor Resection for Metastatic Small Bowel Neuroendocrine Tumors. World J. Surg..

[9] Modica R., Benevento E., Liccardi A., Cannavale G., Minotta R., DI Iasi G., Colao A. (2024). Recent Advances and Future Challenges in the Diagnosis of Neuroendocrine Neoplasms. Minerva Endocrinol..

[10] Rindi G., Mete O., Uccella S., Basturk O., La Rosa S., Brosens L.A.A., Ezzat S., De Herder W.W., Klimstra D.S., Papotti M. (2022). Overview of the 2022 WHO Classification of Neuroendocrine Neoplasms. Endocr. Pathol..

[11] Vahidfar N., Farzanehfar S., Abbasi M., Mirzaei S., Delpassand E.S., Abbaspour F., Salehi Y., Biersack H.J., Ahmadzadehfar H. (2022). Diagnostic Value of Radiolabelled Somatostatin Analogues for Neuroendocrine Tumour Diagnosis: The Benefits and Drawbacks of [^64^Cu]Cu-DOTA-TOC. Cancers.

[12] Fortunati E., Argalia G., Zanoni L., Fanti S., Ambrosini V. (2022). New PET Radiotracers for the Imaging of Neuroendocrine Neoplasms. Curr. Treat. Options Oncol..

[13] Franchina M., Cavalcoli F., Falco O., La Milia M., Elvevi A., Massironi S. (2024). Biochemical Markers for Neuroendocrine Tumors: Traditional Circulating Markers and Recent Development—A Comprehensive Review. Diagnostics.

[14] Lopez-Ramirez F., Yasrab M., Tixier F., Kawamoto S., Fishman E.K., Chu L.C. (2025). The Role of AI in the Evaluation of Neuroendocrine Tumors: Current State of the Art. Semin. Nucl. Med..

[15] Wang S.C., Parekh J.R., Zuraek M.B., Venook A.P., Bergsland E.K., Warren R.S., Nakakura E.K. (2010). Identification of Unknown Primary Tumors in Patients With Neuroendocrine Liver Metastases. Arch. Surg..

[16] Savelli G., Lucignani G., Seregni E., Marchiano A., Serafini G., Aliberti G., Villano C., Maccauro M., Bombardieri E. (2004). Feasibility of Somatostatin Receptor Scintigraphy in the Detection of Occult Primary Gastro-Entero-Pancreatic (GEP) Neuroendocrine Tumours. Nucl. Med. Commun..

[17] Schreiter N.F., Bartels A.-M., Froeling V., Steffen I., Pape U.-F., Beck A., Hamm B., Brenner W., Röttgen R. (2014). Searching for Primaries in Patients with Neuroendocrine Tumors (NET) of Unknown Primary and Clinically Suspected NET: Evaluation of Ga-68 DOTATOC PET/CT and In-111 DTPA Octreotide SPECT/CT. Radiol. Oncol..

[18] Buchmann I., Henze M., Engelbrecht S., Eisenhut M., Runz A., Schäfer M., Schilling T., Haufe S., Herrmann T., Haberkorn U. (2007). Comparison of ^68^Ga-DOTATOC PET and ^111^In-DTPAOC (Octreoscan) SPECT in Patients with Neuroendocrine Tumours. Eur. J. Nucl. Med. Mol. Imaging.

[19] Deppen S.A., Blume J., Bobbey A.J., Shah C., Graham M.M., Lee P., Delbeke D., Walker R.C. (2016). ^68^Ga-DOTATATE Compared with ^111^In-DTPA-Octreotide and Conventional Imaging for Pulmonary and Gastroenteropancreatic Neuroendocrine Tumors: A Systematic Review and Meta-Analysis. J. Nucl. Med..

[20] Kayani I., Bomanji J.B., Groves A., Conway G., Gacinovic S., Win T., Dickson J., Caplin M., Ell P.J. (2008). Functional Imaging of Neuroendocrine Tumors with Combined PET/CT Using ^68^Ga-DOTATATE (DOTA- D Phe^1^,Tyr^3^ -octreotate) and ^18^F-FDG. Cancer.

[21] Bucau M., Laurent-Bellue A., Poté N., Hentic O., Cros J., Mikail N., Rebours V., Ruszniewski P., Lebtahi R., Couvelard A. (2018). 18F-FDG Uptake in Well-Differentiated Neuroendocrine Tumors Correlates with Both Ki-67 and VHL Pathway Inactivation. Neuroendocrinology.

[22] Frilling A., Sotiropoulos G.C., Radtke A., Malago M., Bockisch A., Kuehl H., Li J., Broelsch C.E. (2010). The Impact of ^68^Ga-DOTATOC Positron Emission Tomography/Computed Tomography on the Multimodal Management of Patients With Neuroendocrine Tumors. Ann. Surg..

[23] Prasad V., Ambrosini V., Hommann M., Hoersch D., Fanti S., Baum R.P. (2010). Detection of Unknown Primary Neuroendocrine Tumours (CUP-NET) Using ^68^Ga-DOTA-NOC Receptor PET/CT. Eur. J. Nucl. Med. Mol. Imaging.

[24] Łapińska G., Bryszewska M., Fijołek-Warszewska A., Kozłowicz-Gudzińska I., Ochman P., Sackiewicz-Słaby A. (2011). The Diagnostic Role of ^68^Ga-DOTATATE PET/CT in the Detection of Neuroendocrine Tumours. Nucl. Med. Rev. Cent. East. Eur..

[25] Naswa N., Sharma P., Kumar A., Soundararajan R., Kumar R., Malhotra A., Ammini A.C., Bal C. (2012). ^68^Ga-DOTANOC PET/CT in Patients With Carcinoma of Unknown Primary of Neuroendocrine Origin. Clin. Nucl. Med..

[26] Tan D.S.-W., Montoya J., Ng Q.-S., Chan K.-S., Lynette O., Sakktee Krisna S., Takano A., Lim W.-T., Tan E.-H., Lim K.-H. (2013). Molecular Profiling for Druggable Genetic Abnormalities in Carcinoma of Unknown Primary. J. Clin. Oncol..

[27] Alonso O., Rodríguez-Taroco M., Savio E., Bentancourt C., Gambini J.P., Engler H. (2014). ^68^Ga-DOTATATE PET/CT in the Evaluation of Patients with Neuroendocrine Metastatic Carcinoma of Unknown Origin. Ann. Nucl. Med..

[28] Nakamoto Y., Sano K., Ishimori T., Ueda M., Temma T., Saji H., Togashi K. (2015). Additional Information Gained by Positron Emission Tomography with ^68^Ga-DOTATOC for Suspected Unknown Primary or Recurrent Neuroendocrine Tumors. Ann. Nucl. Med..

[29] Pruthi A., Pankaj P., Verma R., Jain A., Belho E.S., Mahajan H. (2016). Ga-68 DOTANOC PET/CT Imaging in Detection of Primary Site in Patients with Metastatic Neuroendocrine Tumours of Unknown Origin and Its Impact on Clinical Decision Making: Experience from a Tertiary Care Centre in India. J. Gastrointest. Oncol..

[30] Sadowski S.M., Neychev V., Millo C., Shih J., Nilubol N., Herscovitch P., Pacak K., Marx S.J., Kebebew E. (2016). Prospective Study of ^68^ Ga-DOTATATE Positron Emission Tomography/Computed Tomography for Detecting Gastro-Entero-Pancreatic Neuroendocrine Tumors and Unknown Primary Sites. J. Clin. Oncol..

[31] Kazmierczak P.M., Rominger A., Wenter V., Spitzweg C., Auernhammer C., Angele M.K., Rist C., Cyran C.C. (2017). The Added Value of ^68^Ga-DOTA-TATE-PET to Contrast-Enhanced CT for Primary Site Detection in CUP of Neuroendocrine Origin. Eur. Radiol..

[32] Menda Y., O’Dorisio T.M., Howe J.R., Schultz M., Dillon J.S., Dick D., Watkins G.L., Ginader T., Bushnell D.L., Sunderland J.J. (2017). Localization of Unknown Primary Site with ^68^Ga-DOTATOC PET/CT in Patients with Metastatic Neuroendocrine Tumor. J. Nucl. Med..

[33] Sampathirao N., Basu S. (2017). MIB-1 Index–Stratified Assessment of Dual-Tracer PET/CT with ^68^Ga-DOTATATE and ^18^F-FDG and Multimodality Anatomic Imaging in Metastatic Neuroendocrine Tumors of Unknown Primary in a PRRT Workup Setting. J. Nucl. Med. Technol..

[34] Chen S.-H., Chang Y.-C., Hwang T.-L., Chen J.-S., Chou W.-C., Hsieh C.-H., Yeh T.-S., Hsu J.-T., Yeh C.-N., Tseng J.-H. (2018). 68Ga-DOTATOC and 18F-FDG PET/CT for Identifying the Primary Lesions of Suspected and Metastatic Neuroendocrine Tumors: A Prospective Study in Taiwan. J. Formos. Med. Assoc..

[35] Hope T.A., Bergsland E.K., Bozkurt M.F., Graham M., Heaney A.P., Herrmann K., Howe J.R., Kulke M.H., Kunz P.L., Mailman J. (2018). Appropriate Use Criteria for Somatostatin Receptor PET Imaging in Neuroendocrine Tumors. J. Nucl. Med..

[36] Santhanam P., Chandramahanti S., Kroiss A., Yu R., Ruszniewski P., Kumar R., Taïeb D. (2015). Nuclear Imaging of Neuroendocrine Tumors with Unknown Primary: Why, When and How?. Eur. J. Nucl. Med. Mol. Imaging.

[37] Breeman W.A.P., De Jong M., De Blois E., Bernard B.F., Konijnenberg M., Krenning E.P. (2005). Radiolabelling DOTA-Peptides with ^68^Ga. Eur. J. Nucl. Med. Mol. Imaging.

[38] De Dosso S., Treglia G., Pascale M., Tamburello A., Santhanam P., Kroiss A.S., Pereira Mestre R., Saletti P., Giovanella L. (2019). Detection Rate of Unknown Primary Tumour by Using Somatostatin Receptor PET/CT in Patients with Metastatic Neuroendocrine Tumours: A Meta-Analysis. Endocrine.

[39] Navin P.J., Ehman E.C., Liu J.B., Halfdanarson T.R., Gupta A., Laghi A., Yoo D.C., Carucci L.R., Schima W., Sheedy S.P. (2023). Imaging of Small-Bowel Neuroendocrine Neoplasms: *AJR* Expert Panel Narrative Review. Am. J. Roentgenol..

[40] Massimino K.P., Han E., Pommier S.J., Pommier R.F. (2012). Laparoscopic Surgical Exploration Is an Effective Strategy for Locating Occult Primary Neuroendocrine Tumors. Am. J. Surg..

[41] Wang Y.-Z., Chauhan A., Rau J., Diebold A.E., Opoku-Boateng A., Ramcharan T., Boudreaux J.P., Woltering E.A. (2016). Neuroendocrine Tumors (NETs) of Unknown Primary: Is Early Surgical Exploration and Aggressive Debulking Justifiable?. Chin. Clin. Oncol..

